# Photorelaxation and Photorepair Processes in Nucleic and Amino Acid Derivatives

**DOI:** 10.3390/molecules22122203

**Published:** 2017-12-12

**Authors:** Carlos E. Crespo-Hernández

**Affiliations:** Department of Chemistry and Center for Chemical Dynamics, Case Western Reserve University, 10900 Euclid Avenue, Cleveland, OH 44106, USA; cxc302@case.edu

Understanding the fundamental interaction between electromagnetic radiation and matter is essential for a large number of phenomena, with significance to civilization. On the most fundamental level, through the molecular origins of life, photosynthesis, and vision, the interaction between sunlight and matter has played an essential role in nature. Many applications of these interactions continue to revolutionize society through advances in medicine, communications, technology, and entertainment.

Electromagnetic radiation is also capable of inducing a myriad of chemical transformations, as illustrated by the photodegradation of DNA and proteins [1,2,3,4]. These light-induced reactions have been associated with cancer and other diseases in living organisms [5,6,7], and photochemical investigations of nucleic and amino acids continue to be at the forefront of research. Photochemical investigations of modified nucleobases are also at the center of research because of their potential role as prebiotic materials of the building blocks of life [8,9,10] and their prospective applications as phototherapeutic agents [11,12,13]. Studying the basic interactions of these biological molecules with light may hold the key for a complete understanding of the mechanisms responsible for their photostability and photochemistry. It may also provide fundamental insight for a molecular-level understanding of the mechanisms tied to DNA photorepair.

Absorption of ultraviolet or visible radiation by the ground state of a molecule populates electronic states, either directly to an excited singlet state or indirectly to a triplet state after intersystem crossing from the singlet manifold [14]. In both singlet and triplet manifolds, ultrafast internal conversion usually leads to the population of the lowest-energy excited state―the S_1_ and T_1_ states, respectively. These excited states, although relatively short lived (ca. ≤10^−9^ s and ≤10^−6^ s for singlet and triplet states, respectively), may live long enough that chemical reactions can compete with radiative or nonradiative decay to the ground state. Energy or charge transfer from an excited singlet/triplet state of a molecule (a.k.a., sensitizer) may also populate an excited singlet/triplet state of another molecule by a photochemical process known as photosensitization.

In contemporary organic photochemistry, a microscopic description of the electronic relaxation pathways that a photoexcited molecule explores through nuclear coordinate space usually begins with a representation of a reaction coordinate describing the evolution of reactants to products [15,16,17,18,19]. An important distinction between a photochemical and a photophysical relaxation pathway concerns the initial and final states that the reaction coordinate develops. In a photochemical reaction, these states are different, corresponding to the different structures of the reactant(s) and product(s). In a photophysical process, the relaxation pathway ends where it began—in the electronic ground state. In order to return the molecule to its ground state, the absorbed energy can be released radiatively (i.e., through photon emission; usually from the S_1_ or T_1_ state), or it can be transformed into vibrational energy that can dissipate nonradiatively into the environment.

Internal conversion and intersystem crossing can occur at a higher rate than radiative decay when nuclear motions take a molecule into regions of nuclear configuration space where two or more potential energy hypersurfaces cross. The intersection between potential energy hypersurfaces often create crossing seams or conical intersections, where states of equal multiplicity or singlet/triplet crossing regions are involved. The energy gap and the coupling interaction between the electronic and nuclear degrees of freedom largely regulate the rate of the nonadiabatic transitions from one hypersurface to another [15,16,17,19,20].

To thoroughly understand these fleeting photophysical and photochemical processes, a concerted effort from both experimental and theoretical groups is required. Modern quantum-chemical methods and spectroscopic techniques are used to characterize the electronic states, to assign the spectroscopic signatures to specific relaxation pathways or molecular structures, and to delineate the excited-state relaxation mechanisms or photoproduct formation pathways with an unprecedented level of detail.

This Special Issue entitled *Experimental and Computational Photochemistry of Bioorganic Molecules* brings together ten original research articles and two topical perspectives illustrating contemporary developments in the field of nucleic and amino acids photochemistry. It further displays experimental techniques and theoretical methodologies widely used to interrogate the electronic and structural dynamics in these biomolecules. 

Photostability to ultraviolet radiation may have played a key role in the natural selection of the nucleic and amino acid building blocks during the prebiotic era [10,21,22,23], and provides a major driving force for understating photoinduced processes in these biomolecules. Four contributions in this Special Issue investigate the electronic relaxation mechanisms in nucleic and amino acid derivatives. Röttger, Temps, and co-workers [24] examine the excited-state dynamics of the nucleotide xanthosine monophosphate in its neutral and deprotonated forms in aqueous solutions by using a combination of fluorescence up-conversion and transient absorption spectroscopy techniques with femtosecond time resolution. Gustavsson, Markovitsi, and co-workers [25] report on the time-resolved fluorescence decay and fluorescence anisotropy measurements of seven mono-, di-, and tri-methylated xanthine derivatives in water and in methanol. Kohler and co-workers [26] study the excited-state dynamics of melamine (a proto-nucleobase) and its lysine derivatives by using time-resolved infrared spectroscopy. Mališ and Došlić [27] present a comparative computational investigation concerning the excited-state dynamics of three neutral model peptides containing the phenylalanine residue, with a focus on the role that chemical substitution and solvation play on their nonradiative relaxation pathways.

Another major area of focus is to understand the photostability and photochemistry of the canonical nucleobases in isolation and when incorporated in DNA [28,29,30,31,32]. In their Perspective, González and co-workers [33] discuss some of the most crucial challenges associated with the simulation of the excited-state dynamics and product formation in DNA and in its building blocks. The authors summarize the most popular computational methodologies and approximations used in the field and propose alternative quantum-mechanical descriptions that can be used to increase accuracy in the simulation of photoinduced phenomena in these biomolecules. Wang and Chen [34] report on quantum-chemical/molecular-mechanical computations that map the singlet and triplet excited-state relaxation pathways for a stacked conformation between two adjacent thymine nucleobases in DNA oligomers, and compare the results with those obtained for the thymine nucleobase. The goal is to provide a mechanistic understanding for the observation that adjacent thymine nucleobases in DNA are able to minimize photodamage even though thymine–thymine photodimerization can occur in an ultrafast time scale [35,36,37]. Two contributions [38,39] provide new theoretical insights that aim at advancing our understanding of the electronic relaxation pathways in thymine. Segarra-Martí et al. [38] evaluate the effect of electron correlation in the optimization of geometries and in the description of the ^1^ππ* and ^1^nπ* potential energy hypersurfaces of the thymine nucleobase using complete active space second-order perturbation theory. Barbatti and co-workers [39] report on nonadiabatic dynamics simulations for the three lowest-energy excited singlet states of thymine, which are calculated using the algebraic diagrammatic construction to second order method. The authors aim at understanding the role that electron correlation plays in the electronic relaxation pathways of thymine and compare their predictions with experimental results that have been reported in the gas phase.

A prevalent premise of the RNA world hypothesis is that prior to the evolution of more sophisticated cofactors such as flavin adenine dinucleotide, simple and abundant derivatives of purines and pyrimidines may have played the role of a redox coenzyme in RNA-based catalysis [40,41,42]. The primary oxidation product in DNA and RNA, 8-oxo-7,8-dihydro-guanosine, has been proposed as a potential precursor of modern flavin cofactors that may have served as a photorepair agent of cyclobutane pyrimidine dimers in DNA [43]. In their contribution, Wu, Karsili, and Domcke [44] scrutinize this hypothesis computationally by investigating the role of electron-driven proton-transfer reactions in the excited-state relaxation pathways of 8-oxoguanine-adenine and 8-oxoguanine-cytosine base pairs in which 8-oxoguanine is present in its deprotonated (anionic) form. Similarly, heterocyclic oxetanes and azetidines have been suggested as intermediates involved in the photorepair of the thymine-cytosine (6-4) photoproduct by photolyases [45,46], but contrary to the oxetanes, the oxidative and reductive properties of the azetidines have received less attention. In their study, Fraga-Timiraos et al. [47] synthesized two azetidine isomers, which the authors use to model the initial electron transfer step in the photorepair of thymine-cytosine (6-4) photoproducts by photolyases. By performing a series of steady-state and time-resolved fluorescence quenching experiments with photoreductants and photooxidants, the authors evaluate the effect that *cis*, *trans* stereochemistry has on the redox properties of the azetidine isomers and on their electron transfer efficiency.

Sulfur-substituted nucleobases (a.k.a., thiobases) are also endogenous constituents in transfer RNA and may have played an important role in primordial RNA [10,48]. Thiobases and other modified nucleobases provide RNA with additional functions that often broaden their chemical information content [10,49,50]. They have also found wide applications as chemotherapeutic agents [51,52,53]. Two contributions in this Special Issue report on the excited-state dynamics and photochemistry of the thiobases [54,55]. In their Perspective, Arslancan, Martínez-Fernández, and Corral [54] provide an overview about the photophysics and excited-state dynamics of these modified nucleobases from both experimental and computational perspectives. Ashwood et al. [55] investigate the role that N9-glycosylation has on the phototoxic activity of 6-thioguanine—a widely used immunosuppressant and anticancer prodrug [52,56].

All throughout the contributions in this Special Issue, the current state of knowledge regarding the electronic relaxation pathways in nucleic and amino acid derivatives is illustrated. Key experimental tools and computational techniques currently being used for such investigations are presented. It is hoped that the information gathered on these fully open access pages point the way to further developments in the field.
